# Author Correction: High biodiversity in a benzene-degrading nitrate-reducing culture is sustained by a few primary consumers

**DOI:** 10.1038/s42003-021-02311-x

**Published:** 2021-06-18

**Authors:** Chrats Melkonian, Lucas Fillinger, Siavash Atashgahi, Ulisses Nunes da Rocha, Esther Kuiper, Brett Olivier, Martin Braster, Willi Gottstein, Rick Helmus, John R. Parsons, Hauke Smidt, Marcelle van der Waals, Jan Gerritse, Bernd W. Brandt, Wilfred F. M. Röling, Douwe Molenaar, Rob J. M. van Spanning

**Affiliations:** 1 Department of Molecular Cell Biology, AIMMS, Vrije Universiteit Amsterdam, Amsterdam, The Netherlands; 2 Department of Functional and Evolutionary Ecology, University of Vienna, Vienna, Austria; 3 Laboratory of Microbiology, Wageningen University & Research, Wageningen, The Netherlands; 4 Department of Environmental Microbiology, Helmholtz Centre for Environmental Research, Leipzig, Germany; 5 Institute for Biodiversity and Ecosystem Dynamics, University of Amsterdam, Amsterdam, The Netherlands; 6 Unit Subsurface and Groundwater Systems, Deltares, Utrecht, The Netherlands; 7 Department of Preventive Dentistry, Academic Centre for Dentistry Amsterdam, University of Amsterdam and Vrije Universiteit Amsterdam, Amsterdam, The Netherlands

**Keywords:** Environmental microbiology, Microbial ecology

Correction to: *Communications Biology* 10.1038/s42003-021-01948-y, published online 5 May 2021

The original version of the Article contained an error in the y-axis of Figure 3d. The unit of Benzene was incorrectly given as “∝M,” and has been corrected to “μΜ” in the PDF and HTML versions of the Article.

