# Supplementary material for: High biodiversity in a benzene-degrading nitrate-reducing culture is sustained by a few primary consumers
Source: Commun Biol. 2021 May 5;4:530. doi: 10.1038/s42003-021-01948-y (PMC8099898; doi:10.1038/s42003-021-01948-y)
Supplement: Supplementary file 3 — Description of Additional Supplementary Files [file 42003_2021_1948_MOESM3_ESM.pdf]

Description of Additional Supplementary Files

File name: Supplementary Data 1

Description: Basic genome information, quality and relative RNA and DNA abundances of all MAGs.

File name: Supplementary Data 2

Description: Environmental variables measured throughout the succession experiment. Cell No: log 10 of the cell number (see Supplementary Methods 1.5 - 1.7 for details).
