# Supplementary material for: High biodiversity in a benzene-degrading nitrate-reducing culture is sustained by a few primary consumers
Source: Commun Biol. 2021 May 5;4:530. doi: 10.1038/s42003-021-01948-y (PMC8099898; doi:10.1038/s42003-021-01948-y)
Supplement: Supplementary file 6 — Reporting Summary [file 42003_2021_1948_MOESM6_ESM.pdf]

nature research

Corresponding author(s): Chrats Melkonian and Rob van Spanning

Last updated by author(s): Sep 1, 2020

Reporting Summary

Nature Research wishes to improve the reproducibility of the work that we publish. This form provides structure for consistency and transparency in reporting. For further information on Nature Research policies, see our Editorial Policies and the Editorial Policy Checklist.

Statistics

For all statistical analyses, confirm that the following items are present in the figure legend, table legend, main text, or Methods section.

n/a	Confirmed	
☐	☒	The exact sample size (*n*) for each experimental group/condition, given as a discrete number and unit of measurement
☐	☒	A statement on whether measurements were taken from distinct samples or whether the same sample was measured repeatedly
☐	☒	The statistical test(s) used AND whether they are one- or two-sided<br>*Only common tests should be described solely by name; describe more complex techniques in the Methods section.*
☒	☐	A description of all covariates tested
☐	☒	A description of any assumptions or corrections, such as tests of normality and adjustment for multiple comparisons
☐	☒	A full description of the statistical parameters including central tendency (e.g. means) or other basic estimates (e.g. regression coefficient) AND variation (e.g. standard deviation) or associated estimates of uncertainty (e.g. confidence intervals)
☐	☒	For null hypothesis testing, the test statistic (e.g. *F*, *t*, *r*) with confidence intervals, effect sizes, degrees of freedom and *P* value noted<br>*Give P values as exact values whenever suitable.*
☒	☐	For Bayesian analysis, information on the choice of priors and Markov chain Monte Carlo settings
☒	☐	For hierarchical and complex designs, identification of the appropriate level for tests and full reporting of outcomes
☒	☐	Estimates of effect sizes (e.g. Cohen's *d*, Pearson's *r*), indicating how they were calculated

Our web collection on statistics for biologists contains articles on many of the points above.

Software and code

Policy information about availability of computer code

Data collection	No software, open-source or custom code used to collect data.
Data analysis	Code and processed data/tables can be found in: https://github.com/SystemsBioinformatics/anaerobic-benzene-degrading-culture.<br>Metagenome-assembled genomes can be found in an Anvi'o result at zenodo open-access repository under http://doi.org/10.5281/zenodo.3939224

For manuscripts utilizing custom algorithms or software that are central to the research but not yet described in published literature, software must be made available to editors and reviewers. We strongly encourage code deposition in a community repository (e.g. GitHub). See the Nature Research guidelines for submitting code & software for further information.

Data

Policy information about availability of data

All manuscripts must include a data availability statement. This statement should provide the following information, where applicable:

- Accession codes, unique identifiers, or web links for publicly available datasets
- A list of figures that have associated raw data
- A description of any restrictions on data availability

Metagenomics shotgun & 16S rRNA gene amplicon sequencing are available in the European Nucleotide Archive under primary accession PRJEB39357.

Field-specific reporting

Please select the one below that is the best fit for your research. If you are not sure, read the appropriate sections before making your selection.

☐ Life sciences ☐ Behavioural & social sciences ☒ Ecological, evolutionary & environmental sciences

For a reference copy of the document with all sections, see nature.com/documents/nr-reporting-summary-flat.pdf

Ecological, evolutionary & environmental sciences study design

All studies must disclose on these points even when the disclosure is negative.

Study description	We studied diversity and niche partitioning of a microbial community with more than 100 species evolved in a bioreactor running for 15 years with benzene as the main carbon and free energy source and nitrate as the electron acceptor.
Research sample	-Samples were gathered from biofilms formed within the reactor by scraping off a confined area of biofilms with two distinct morphologies referred to as brown and white biofilm samples.<br>-To study succession of the microbial community, we inoculated 96 anoxic bottles from which we sacrificed 12 bottles each at time points (2h, 7 days, 14 days, 18 days, 22 days, 26 days, 30 days and 34 days after inoculation), and used for microbial community analysis.
Sampling strategy	-From the 15-years old bioreactor, triplicate biofilm samples were taken for the white and brown biofilm types, under constant flow of sterile nitrogen.<br>-From the succession experiment, a pilot experiment was performed to determine general trends of benzene consumption over time by taking quadruplicate samples under the same conditions of the final succession experiment.
Data collection	-From the 15-years old bioreactor: the extracted DNA and RNA samples were used for genome-resolved metagenomics and metatranscriptomics sequencing, respectively. Benzene and nitrate were measured regularly using GC-FID and HPLC methods, respectively.<br>-From the succession experiment: 16S rRNA gene amplicon sequencing, flow cytometry, LC-MS/MS, capillary electrophoresis
Timing and spatial scale	-From the 15-years old bioreactor samples were taken at a single time point, no time series were made. The white biofilm samples were taken on 31-10-2014 and the brown biofilm samples on 3-11-2014. The rational for sampling in different dates was to avoid extensive exposure of the anaerobic bioreactor community to oxygen.<br>-From the succession experiment samples were taken at intervals of a few days (as indicated) up to 34 days maximum. The experiment started on 15-02-2014 and ended on 21-03-2014.
Data exclusions	-From the 15-years old bioreactor, one biofilm sample was a partial mixture of both liquid and biofilm phases and was not included for further analyses.<br>-From the succession experiment, the DNA from 9 out of the 12 bottles at the first sampling time point (2h) were too low and were not included for further analyses.
Reproducibility	Multiple analytical techniques were used for the validity of the experimental data
Randomization	- For the 15-years old bioreactor randomization was not applicable given the low number of samples<br>- For the succession experiment randomization was not applicable. This was a controlled experiment with identical and identically treated samples with no obvious covariates.
Blinding	- Not applicable to the 15-years old bioreactor sampling. The samples picked for DNA and RNA extraction was based on the morphology of their biofilm samples. Therefore, sample selection was not Blind.<br>- Not applicable to the succession experiment that was fully controlled.

Did the study involve field work? ☐ Yes ☒ No

Reporting for specific materials, systems and methods

We require information from authors about some types of materials, experimental systems and methods used in many studies. Here, indicate whether each material, system or method listed is relevant to your study. If you are not sure if a list item applies to your research, read the appropriate section before selecting a response.

Materials & experimental systems

n/a	Involved in the study
[x]	[ ] Antibodies
[x]	[ ] Eukaryotic cell lines
[x]	[ ] Palaeontology and archaeology
[x]	[ ] Animals and other organisms
[x]	[ ] Human research participants
[x]	[ ] Clinical data
[x]	[ ] Dual use research of concern

Methods

n/a	Involved in the study
[x]	[ ] ChIP-seq
[ ]	[x] Flow cytometry
[x]	[ ] MRI-based neuroimaging

Flow Cytometry

Plots

Confirm that:

- [ ] The axis labels state the marker and fluorochrome used (e.g. CD4-FITC).
- [ ] The axis scales are clearly visible. Include numbers along axes only for bottom left plot of group (a 'group' is an analysis of identical markers).
- [ ] All plots are contour plots with outliers or pseudocolor plots.
- [ ] A numerical value for number of cells or percentage (with statistics) is provided.

Methodology

Sample preparation	In a 15 mL polypropylene tube (Greiner, Sigma-Aldrich Co.,Zwijndrecht, The Netherlands) we aliquot 2 mL of cell suspension. Cells were fixed by addition of 50 mL of formamide to each cell suspension, this suspension was inverted 10 times gently and incubated it 10 min at room temperature. After, we added 4 mL of 10 mM Sodium Pyrophosphated dissolved in filtered and sterilized (autoclave, 121C for 15 min) Ultrapure water from a Barnstead Nanopure Diamond (Beun De Rond, The Netherlands). Aggregates of cells were disassembled by sonication by placing the polypropylene tubes in a plastic glass filled with water, no more than four at a time. We optimized sonication conditions by varying the amplitude, time and interval of sonication using a SONIPREP 150 Ultrasonic Disintegrator (MSE Ltd., London, UK). Optimal sonication was determined by light microscopy of cell suspensions prior and after sonication and consisted of 3x sonication with 30 sec interval, when tubes were softly inverted, using 15 mm of amplitude. We stained cells by adding of 0.5 mL of SYBR Green II (Thermo Fisher Scientific Inc., Reinach, Switzerland), inverting tubes ten times and incubating them for 5 min in the dark. Prior flow cytometry, we fixed, sonicated and stained 2 mL of the same batch of non-inoculated benzene medium used in the succession experiments to measure the presence of aggregates that could be stained by SYBR-Green II. Cell densities, expressed as numbers of cells per mL, were determined by running 200 mL of cells suspensions and non-inoculated benzene medium
Instrument	Accuri C6 Flow Cytometer System (Accuri Ctometers, Ltd., Cambs, UK) using medium velocity setting and Filter FL1-A (specific for SYBR-Green).
Software	CFlow Plus software (CFlow) was used to control the C6 Flow Cytometer® system and acquire data, generate statistics, and analyze results.
Cell population abundance	Not applicable for this experiment: We only used flow cytometry to obtain total cell counts.
Gating strategy	Not applicable for this experiment: We only used flow cytometry to obtain total cell counts.

- [ ] Tick this box to confirm that a figure exemplifying the gating strategy is provided in the Supplementary Information.
